# Comparison of the effectiveness of virtual reality-based education and conventional teaching methods in dental education: a systematic review

**DOI:** 10.1186/s12909-023-04954-2

**Published:** 2024-01-03

**Authors:** Hossain Koolivand, Mohammad Mahdi Shooreshi, Roya Safari-Faramani, Milad Borji, Meysam Siyah Mansoory, Hedaiat Moradpoor, Masoud Bahrami, Seyyed Mohsen Azizi

**Affiliations:** 1 Dental Students’ Research Committee, School of Dentistry, Kermanshah University of Medical Sciences, Kermanshah, Iran; 2 Dental Students’ Research Committee, School of Dentistry, Tehran University of Medical Sciences, Tehran, Iran; 3 Assistant Professor of Epidemiology Department of Epidemiology, School of Health Research Center for Environmental Determinants of Health Research Institute, Kermanshah University of Medical Sciences, Kermanshah, Iran; 4 Faculty Member, Department of Nursing, Kermanshah University of Medical Sciences, Kermanshah, Iran; 5 Faculty Member, Department of Biomedical Engineering, School of Medicine, Kermanshah University of Medical Sciences, Kermanshah, Iran; 6 Associate Professor in Prosthodontics, Department of Prosthodontics, School of Dentistry, Kermanshah University of Medical Sciences, Kermanshah, Iran; 7 Research assistance, Arak University of Medical Sciences, Arak, Iran; 8 Medical Education and Development Center, Arak University of Medical Sciences, Arak, Iran

**Keywords:** Dental education, Virtual reality, Conventional teaching methods, Systematic review

## Abstract

**Background and objectives:**

Virtual reality dental simulators as an educational tool may create specific training conditions for dental students, allowing them to practice dental skills in a safe and controlled condition. This study aimed to investigate the effectiveness of virtual reality-based education in dental education compared to traditional education approaches.

**Methods:**

In this systematic review, four databases (PubMed, Scopus, Web of Science, and Science Direct) were searched until 2023 following PRISMA guidelines. The Quality assessment and risk of bias were assessed by the Cochrane Collaboration Tool for RCTs and GRADE, respectively. Inclusion criteria were restricted to randomized or quasi-randomized trials about virtual reality efficacy in dental education. Two authors independently evaluated the data and reviewed the overall risk of bias for all selected studies. Study design, sample size, hardware, onset time of intervention, duration, and number of procedures performed were among the data extracted.

**Results:**

From the 703 titles, 48 full texts were chosen for review, yielding 14 articles for final inclusion. The review of these articles indicated the effective role of virtual reality dental simulators in improving students' knowledge and practical skills.

**Conclusions:**

Based on our findings, adding haptic technology to virtual reality can improve students' practical skills, hand skills, theoretical knowledge, self-confidence, and learning environment. Although a fair amount of research needs to be done, notably on cost-effectiveness, student satisfaction, and other potentially adverse effects, virtual reality is a growing phenomenon with immense potential.

## Background

Virtual reality (VR) is a three-dimensional (3D) artificial simulation of a real-life environment or situation in computer systems first used in dental education in 1988 [1]. It allows users to interact with the virtual reality environment by simulating vision and audience in real-time. VR technology is based on three main principles immersion, interaction, and user intervention in the virtual reality environment. Immersion shows the presence in the virtual environment, and interaction indicates the operator's modification performance [2]. VR types of dentistry equipment are camera-display systems or head-mounted systems [3].

Some studies show that although dental students acquire sufficient knowledge and skills in preclinical courses, their education process has challenges and limitations [4, 5]. Dentistry education differs from education in other health fields due to the combination of theoretical, practical-laboratory topics and clinical exercises. Spatial imagination to acquire theoretical knowledge in dentistry is one of the essential requirements in dental education. This requirement may not be fulfilled in traditional learning environments. Therefore, using the capabilities of new technologies such as virtual reality may be an effective solution to improving the quality of dental education. VR is used as a complementary tool in teaching practical skills to dental students before facing real patients [6–8]. VR can eliminate many limitations of traditional education. VR increases the ability of dental students in self-assessment and self-learning, it does not have limitations related to the time frame of practice in dental laboratories, and it provides an endless and timeless opportunity for practice and learning [9, 10]. This technology enables students to practice in a very low-risk environment [11], reducing costs in the long run [9]. Also, VR technology reduces students' need for a teacher (the teacher is a facilitator and observer) [9] and makes the learning process more standardized [10, 12].

Studies have been conducted in recent years on VR technology's effect on dental students' performance. Despite the emphasis of studies on the effectiveness of VR-based education, different results have been reported in some studies [13–15]. A systematic review of studies conducted in dentistry to understand the effect of teaching designed using VR dental simulators in comparison with conventional teaching on improving students' theoretical knowledge and practical skills can provide new insights into the role of VR technologies in the teaching and learning process. This systematic review aimed to compare the effectiveness of VR-based education and conventional teaching methods for dental sciences to determine whether VR can improve dental students' learning performance. About the aim of the study, the main question is: What are the advantages of implementing simulators based on VR in dental education compared to traditional methods for enhancing the student's knowledge and learning motor skills?

## Materials and methods

This systematic review protocol was conducted according to the Preferred Reporting Items for Systematic Reviews and Meta-Analyses (PRISMA) [16]. To decrease bias, the process of searching, selection of articles, evaluation of the quality of the studies, and extraction of data was done by two independent researchers, and if needed, the third author judged any disagreements. With the help of the quality assessment tool, the quality of each article was evaluated independently by two of the authors.

### Search strategy

**Fig. 1:**
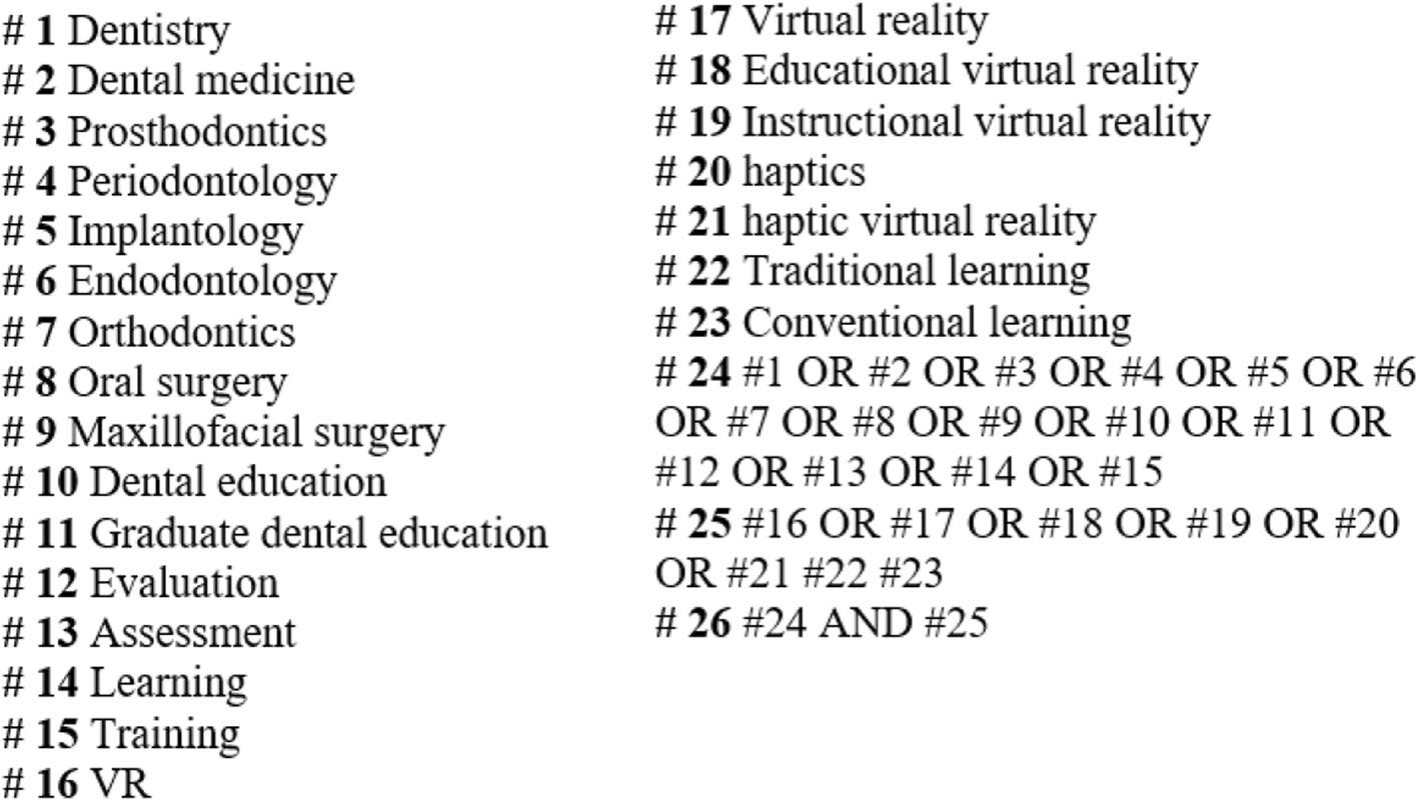
Search strategy in database

A systematic search was performed in PubMed, Scopus, Web of Science, and Science Direct databases up to September 2023 for articles in English-language journals. The search strategy is shown in Fig. 1.

This study used the PICOS tool, which focuses on 5 indicators population, Intervention, Comparison, Outcomes, and Study design. Population (P): The population index was related to undergraduate and graduate dental students. Intervention (I): The intervention included educational methods based on virtual reality technology. Comparison (C): This index included comparing methods based on virtual reality and conventional/traditional methods. Outcome (O): The result included the effect of virtual reality-based educational methods on the acquisition of knowledge and skills of dental students. Study design (S): The study design focused on randomized controlled and quasi-randomized trials.

The following MeSH-related English keywords were used: virtual reality, haptics, haptic virtual reality, virtual reality environment, instructional virtual reality, educational virtual reality, dental education, graduate dental education, and continuing dental education, traditional teaching/learning/training, conventional teaching/learning/training. In addition, the combination of these words was used using AND-OR operators.

### Inclusion and exclusion criteria

The inclusion criteria were randomized controlled trials, and quasi-randomized trials only in the selected period, examination of at least one human subject related to the topic of VR, information available on the VR technology used and its association with the dental discipline, presence of a VR test group and a traditional learning control group. The exclusion criteria were case–control studies, review studies, articles comparing two virtual reality groups with no traditional control group, studies that reported incomplete information, studies conducted on patients and not students, and studies evaluating a simulator's effect.

### Data extraction

To extract data from the articles, a checklist was used that included information about the author's name, the year of publication of the study, the sample size, the type of virtual reality tool, the duration of the intervention, and the results. The results were classified using Microsoft Excel 2019 (Microsoft Corporation, Redmond, WA, United States) and EndNote X6 software (Thomson Reuters EndNote X6.1.0).

### Quality assessment

The methodological quality of each article was evaluated independently by two authors. the risk of bias assessment was performed in each study using the Cochrane collaboration tool for RCTs and based on the following items [17] (oralhealth.cochrane.org) (See Table 1): Selection bias, performance, and detection bias, bias due to incomplete data, reporting bias, and other biases (including industry sponsorship bias).

**Table 1:** Presentation of risk of bias evaluation for included studies according to the cochrane collaboration's tool

	Random sequence generation	Allocation concealment	Blinding of participants	Blinding of outcome assessment	Incomplete outcome data	Selective reporting	Other bias
Murbay et al. [18]	Unclear risk of bias	Unclear risk of bias	Low risk of bias	Low risk of bias	Low risk of bias	Low risk of bias	Low risk of bias
Dwisaptarini et al. [19]	Low risk of bias	Low risk of bias	Low risk of bias	Low risk of bias	Low risk of bias	Low risk of bias	Low risk of bias
Pulijala et al. [20]	Low risk of bias	Unclear risk of bias	High risk of bias	Low risk of bias	High risk of bias	Low risk of bias	Low risk of bias
Tubelo et al. [21]	Low risk of bias	Low risk of bias	High risk of bias	Low risk of bias	High risk of bias	Low risk of bias	Low risk of bias
Samuel Koo et al. [22]	Low risk of bias	Low risk of bias	High risk of bias	Unclear risk of bias	Unclear risk of bias	Low risk of bias	Low risk of bias
Hirono Kikuchi et al. [23]	Unclear risk of bias	Unclear risk of bias	Low risk of bias	Low risk of bias	Low risk of bias	Low risk of bias	Low risk of bias
Riki Gottlieb et al. [24]	Unclear risk of bias	Unclear risk of bias	Unclear risk of bias	High risk of bias	Low risk of bias	Low risk of bias	Low risk of bias
Nardy Casap et al. [25]	Low risk of bias	Unclear risk of bias	High risk of bias	Low risk of bias	Low risk of bias	Low risk of bias	Low risk of bias
Suebnukarn et al. [26]	Low risk of bias	Low risk of bias	Low risk of bias	Low risk of bias	Low risk of bias	Low risk of bias	Low risk of bias
Buchanan et al. [27]	Unclear risk of bias	High risk of bias	Unclear risk of bias	Low risk of bias	Low risk of bias	Low risk of bias	Low risk of bias
Al-Saud et al. [28]	Low risk of bias	Unclear risk of bias	Low risk of bias	Low risk of bias	Low risk of bias	Low risk of bias	Low risk of bias
Liebermann et al. [14]	Low risk of bias	Unclear risk of bias	Unclear risk of bias	High risk of bias	Low risk of bias	Low risk of bias	Low risk of bias
Zhang et al. [29]	Low risk of bias	Low risk of bias	Unclear risk of bias	Low risk of bias	Low risk of bias	Low risk of bias	Low risk of bias
SiahMansoory et al. [13]	Low risk of bias	Low risk of bias	Low risk of bias	Low risk of bias	Low risk of bias	Low risk of bias	Low risk of bias

Another tool to measure the quality of articles in this study was GRADE (Grading of Recommendations Assessment, Development, and Evaluation). GRADE is a tool that can be used to evaluate and rank the quality of studies in four levels: very low, low, moderate, or high. Based on this tool, studies are evaluated regarding risk of bias, inconsistency, indirectness, imprecision, or publication bias. The results of the evaluation of the studies are presented in Table 2.

**Table 2:** Assessment of the included studies using the GRADE scale

Author / Year	Risk of bias	Imprecision	Inconsistency	Indirectness	Publication Bias
Murbay et al. [18]	Very low	Very low	Very low	Very low	Very low
Dwisaptarini et al. [19]	Very low	Very low	Very low	Very low	Very low
Pulijala et al. [20]	Very low	Very low	Very low	Very low	Very low
Tubelo et al. [21]	Very low	Very low	Very low	Very low	Very low
Samuel koo et al. [22]	Very low	Very low	Very low	Very low	Very low
Hirono Kikuchi et al. [23]	Very low	Very low	Very low	Very low	Very low
Riki Gottlieb et al. [24]	Very low	Very low	Very low	Very low	Very low
Nardy Casap et al. [25]	Very low	Very low	Very low	Very low	Very low
Suebnukarn et al. [26]	Very low	Very low	Very low	Very low	Very low
Buchanan et al. [27]	Very low	Very low	Very low	Very low	Very low
Al-Saud et al. [28]	Very low	Very low	Very low	Very low	Very low
Liebermann et al. [14]	Very low	Very low	Very low	Very low	Very low
Zhang et al. [29]	Very low	Very low	Very low	Very low	Very low
Siah Mansoory et al. [13]	Very low	Very low	Very low	Very low	Very low

**Fig. 2:**
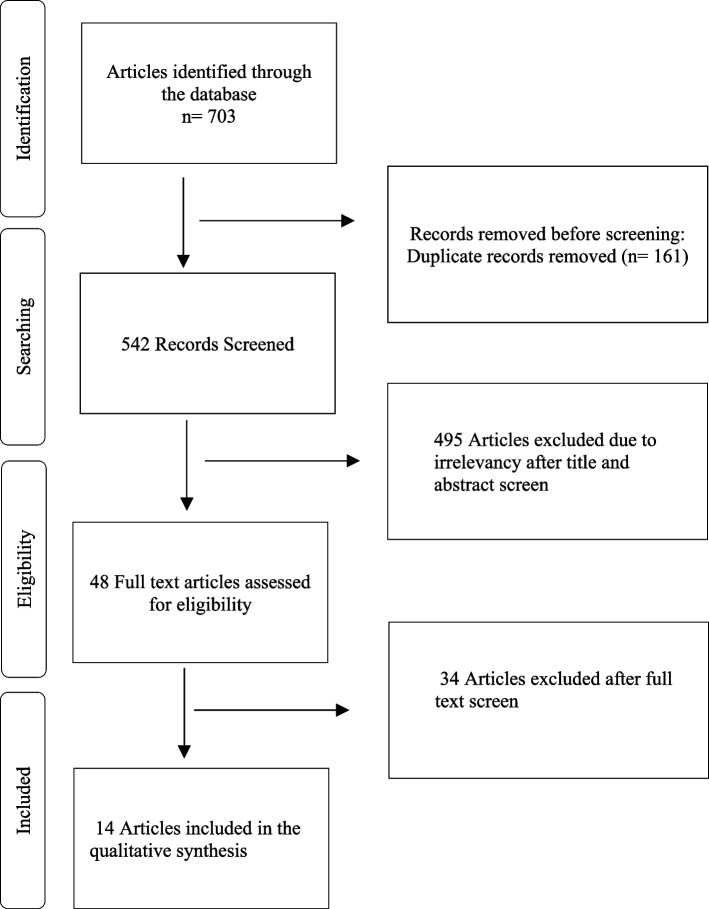
Flowcharts of the study and selection of articles based on PRISMA steps

As shown in Fig. 2, The number of 703 articles were retrieved in the primary search, which decreased to 542 articles after removing the duplicates. After reviewing the titles and abstracts, 48 articles were selected for full-text evaluation. Finally, 14 eligible articles were included in the study. After evaluating the full-text of the articles, 34 articles were excluded due to lack of inclusion criteria.

Reasons for exclusion: articles lacking sufficient information about the use of virtual reality technology (*n* = 10), comparing two groups of virtual reality (*n* = 7), narrative reviews and/or opinion letters (*n* = 8), experimental laboratory research (*n* = 5), and studies conducted on patients (*n* = 4).

### Ethical considerations

This study was approved by the Ethics Committee of Kermanshah University of Medical Sciences (KUMS) (IR.KUMS.REC.1398.1244).

## Results

Table 3 provides a summary of the information on the articles included in the study. The analysis of the articles indicated that 36% of the articles are from American countries (The United States, Brazil) [22, 24, 25, 27, 30], 36% from Asian countries (Thailand, Japan, Iran, and China) [13, 19, 23, 26, 31], and 28% from European countries (the United Kingdom, Ireland, and Germany) [18, 20, 28, 32]. In these studies, dental students from different levels have been studied. The educational goal of the studies was to focus on improving the level of knowledge and skills of dental students.

**Table 3:** Summary of findings across 14 studies

Article/number	14
Article per country	America (*n* = 5, 36%), Asia (*n* = 5, 36%), Europe (*n* = 4, 28%)
Total number of students	628
Average number of students per study	48
Level of education	First-year (16); Sophomores and Juniors (94); Fourth-year (32); Fifth-year (43); Sixth-year (85); Final-year (40); Undergraduate (141); Preclinical (82); Residents (95)
The educational objective of each study	Knowledge (4); Skill (8); Knowledge and Skill (2)

Table 4 presents comprehensive information on the included studies. It contains a list of retrieved articles, equipment used, methodology, sample size, and a brief description of the results. Because of the heterogeneity in the different dental interest fields, validated comparisons between the selected publications were not possible, and no meta-analysis could be performed. All of the included studies were in the field of dental education. The reviewed studies included a diverse range of topics from training in simple tooth cavity preparation to complex surgical training approaches. Studies have evaluated the effectiveness of using virtual reality simulators as an educational tool, especially in preclinical knowledge acquisition.

**Table 4:** List of retrieved articles along with the equipment used, methodology, sample size, and brief description of their results

	**Author**	**Test Group**	**Control group**	**Equipment used**	**Dental procedures involved**	**Results**	**Key findings**
1	Murbay et al. [18]	Sixteen 2nd year students	Sixteen 2nd year students	Moog Simodont	Tooth preparation	The percentage of satisfactory domains was significantly higher in group 1, compared to group 2, both in the manual evaluation (83.9% (94/112) and 59.8% (67/112) in groups 1 and 2, respectively) and in the digital evaluation (85.7% (96/112) and 55.4% (62/112) in groups 1 and 2, respectively) (*P* < .05)	The use of VR significantly improved the satisfactory performance of students. The virtual reality simulator may be a valuable adjunct in the undergraduate direct restoration course and for student remedial
2	Dwisaptarini et al. [19]	Sixteen 6th-year students	Sixteen 6th-year students	Two omni haptic devices (sensAble Inc., Woburn, MA, USA)	Caries removal	The equivalence test for proportional differences (two 1-sided t-tests) with a 0.2 margin confirmed that the participants in both groups had identical post-training performance scores (95% CI = 0.92, 1; *p* = 0.00)	Training on the micro-CT multi-layered caries model with the visuo-tactile virtual reality simulator and conventional extracted tooth had equivalent effects on improving the performance of minimally invasive caries removal
3	Pulijala et al. [20]	51 freshmen postgraduate students	44 freshmen postgraduate students	Oculus Rift and Leap Motion	Le Fort I osteotomy	Comparing the relative improvement in the confidence levels, the participants of the study group showed significantly higher self-confidence scores than those in the control group (F = 4.63, *p* = 0.034)	Immersive Virtual Reality experiences improve the knowledge and self-confidence of the surgical residents
4	Tubelo et al. [21]	Two groups of 9 and 15 students	Two groups of 9 students	Not mentioned	Cementation	The theoretical posttest showed a significant difference between the longitudinal groups, GLC (6.0 ± 1.15) and GLVLO (7.33 ± 1.43). The lower film thickness presented with a significant difference in the VLO groups: (GIC 25 ± 9.3) and GIVLO (16.24 ± 5.17); GLC (50 ± 27.08) and GLVLO (22.5 ± 9.65). The higher setting time occurred in the VLO groups, and the immediate group showed a significant difference (GIC 896 ± 218.90) and GIVLO (1138.5 ± 177.95)	The groups that used the virtual learning object (VLO) had superior clinical handling skills to controls and greater retention of knowledge after 15 days. The use of VLO in Dentistry could enhance continual educational programs increasing the quality of health assistance
5	Koo et al. [22]	17 dental students	17 dental students	Haptic device and IDEA software	Cavity preparation	Improvement of overall tooth preparation scores post-haptic use was not statistically significant compared to controls (*P* > 0.05). However, students found the game feature of the haptic device made the learning experience more fun and interesting	The haptic exercises with the manual dexterity module software were not superior in improving the dexterity of students for tooth cavity preparations in the short term. The benefits of ease of use and fun learning experience can be further investigated in future studies
6	Hirono Kikuchi et al. [23]	Thirteen 5th year students	Thirteen 5th year students	DentSim	Crown preparation	The total scores of students in the DSF and DS groups were significantly higher than those in the NDS group (*P* < 0.05), 69 and 60 vs. 10	The results of this study suggested that the use of the VRS system improved student training for PFM crown preparation
7	Riki Gottlieb et al. [24]	12 faculty members to assess 97 first-year students	12 faculty members to assess 97 first-year students	DentSim	Theoretical and practical education	Faculty perceptions of VRS students' abilities were higher than those of non-VRS students for most abilities examined. However, the faculty members' expectations of VRS training were higher than their perceptions of the student's abilities after VRS training for most abilities examined (*P* < 0.05)	Ergonomic development and technical performance were positively impacted by virtual reality simulation (VRS) training. These results support the use of VRS in a preclinical dental curriculum
8	Casap et al. [25]	20 senior students	20 senior students	Navigation system (Denex image-guided implantology [IGI]; DenX advanced dental systems; Moshav ora)	Implant placement	The execution of all assignments was significantly faster in the freehand group than in the navigation group (60.75 vs. 77.25 min, *P* = .02)	Despite the improved performance of the navigation system, the added value of training in dental implantation surgery with virtual reality navigation was minimal
9	Suebnukarn et al. [26]	Sixteen 4th year students	Sixteen 4th year students	Haptic (SensAble Inc., Woburn, MA, USA) VR simulator & micro-CT tooth models	Access cavity preparation	Post-training performance had improved compared with pre-training performance in error scores in both groups (*P* < 0.05). However, the error score reduction between the haptic VR simulator and the conventional training group was not significantly different (*P* > 0.05). The VR simulator group decreased significantly (*P* < 0.05) the amount of hard tissue volume lost during the post-training exercise. Task completion time was not significantly different (*P* > 0.05) in both groups. The total score of post-training error scores between haptic virtual reality (VR) training and phantom head training groups were 3.78 ± 1.10 and 3.98 ± 1.41 respectively	Training on the haptic VR simulator and conventional phantom head had equivalent effects on minimizing procedural errors in endodontic access cavity preparation. The results suggested great promise of haptic VR and micro-CT tooth models as a tool for endodontic access cavity preparation training
10	Buchanan et al. [27]	First study: 8 first-year students Second study: 14 first-year students	First study: 8 first-year students Second study: 14 first-year students	DentSim	Cavity preparation	The scores of practical examinations in the control and experimental groups were 79.3 and 72 respectively and were significant (*P* < 0.05)	The study concludes that VR technology offers significant potential in the field of dental education and that further use and investigation are both desired and justified
11	Al-Saud et al. [28]	Sixty-three with no previous dental training	-	Simodont haptic dental simulator	Manual dexterity exercises from the Courseware package	The overall composite error scores were significantly different amongst the Groups [F (2, 60) = 5.63, *P* = 0.006, g2 *p* = 0.158]. There were no significant differences amongst groups in the total time taken to perform the task (drill time) during all training exercises, [F (2.52, 151) = 1.078, *P* = 0.4, g2 *p* = 0.018]. However, significant main differences amongst the groups in the task completion percentage (i.e. how much of the target zone was removed) were found, [F (3.6, 109) = 7.06, *P* = 0.001, g2 *p* = 0.19]. Post hoc analysis revealed that the DFB group had significantly higher TC scores than other groups in the first (*P* = 0.001) and the fourth (*P* = 0.004) training exercises	The study conclusions indicate that the acquisition and retention of basic dental motor skills in novice trainees is best optimized through a combination of instructor and visual display (VR)–driven feedback
12	Liebermann et al. [14]	82 First-semester preclinical students	82 First-semester preclinical students	Oculus Quest 2 All-In-One (Menlo Park, CA, USA; RAM (Random-access memory) memory: 6 GB; Internal storage capacity: 64 GB)	Teeth morphology training (assessed by 2 theoretical tests)	By differentiating the two student groups (use of VR glasses for anterior/posterior teeth) within the dental experience group, significantly better test results (*p* = 0.040) were shown for group 1 in the total posterior teeth test score. Furthermore, no other significant differences, but a possible tendency, in the test results and thus no effect of the use of the VR glasses on both VR groups could be analyzed (*p* ≥ 0.051)	1. Additional learning of tooth morphology in the VR tooth learning environment did not improve anterior or posterior teeth recognition test outcomes. 2. Anterior teeth test scores were significantly better than posterior teeth test scores in teeth recognition and tooth characteristics. 3. In test group 2, students with dental professional experience performed better on the test, with statistically significant disparities
13	Zhang et al. [29]	30 (2*15) Second- and third-year undergraduate students pursuing Stomatology at Lanzhou University. (Groups J and V)	30 (2*15) Second and third-year undergraduate students pursuing Stomatology at Lanzhou University. (Groups V-J and J-V)	UniDental	periodontal theoretical and operational skills (scaling process)	The findings showed no significant difference in the first theoretical outcomes between the four groups (*P* > 0.05). The scores of the second theoretical assessment were significantly improved for the V-J and J-V groups (60.00 ± 4.47, 58.33 ± 4.35) compared with the scores of the first theoretical exam (49.67 ± 4.81, 48.00 ± 4.93, *P* < 0.05). The operation process scores of students in Group V-J and J-V (72.00 ± 5.92; 70.00 ± 3.05) were significantly higher compared with the scores in the other two groups (V: 61.67 ± 7.85; J: 60.67 ± 2.58). The scaling process performance of students in Group V-J and J-V (53.00 ± 3.05; 63.40 ± 4.39) was improved compared with that of students in the other two groups (V: 41.90 ± 5.23; J: 47.40 ± 4.31)	Combining VR and a jaw model during periodontal preclinical training can enhance the students' grades and significantly improve professional skills. To maximize learning in basic periodontal education, the jaw model should be used before VR. This work provides a foundation for future periodontal preclinical training strategies
14	SiahMansoory et al. [13]	25 6th-year students	25 6th-year students	VR headset, EKEN 4 K UHD 60	Neutral zone, Teeth arrangement	The majority of students (76%) were highly satisfied with the use of VR technology in their learning process. The mean score of students was significantly higher in the VR group (16.92 ± 1.12) than in the lecture group (16.14 ± 1.18)	VR technology is useful and effective in the teaching–learning process. Therefore, its use in medical and dental schools can play an effective role in creating a dynamic, attractive, and successful learning environment

The results showed the implementation of VR technologies in dental education helps various fields. Each field needs different techniques and methods for using virtual reality technologies in the learning process. Among the 14 articles, VR technologies help dental education in restorative dental procedures, tooth preparation [22], caries removal [19], access cavity preparation [22], implant treatment [25], crown preparation [27], Le Fort I osteotomy [20], neutral zone and Teeth arrangement assessment [13], and cementation [30].

A systematic review of studies in terms of the effect of VR simulators in dental education indicates an improvement in students' academic performance. The improvement in students' academic performance was evident in both theoretical knowledge and practical skills levels. The results of the reviewed studies are presented separately in Table 4.

The evaluated studies examined a variety of learning aspects, such as preparation, transition, and retention, to determine the effectiveness of VR in theoretical knowledge learning. Based on the review conducted in this study, the theoretical knowledge of dental students can be improved through the use of VR technologies.

In addition to the mentioned cases, the findings show that the addition of VR to more traditional teaching methods has formed newly recognized student-centered teaching methods. In this connection, studies reported that the use of VR might affect certain learning elements such as preparation, transition, and retention [28, 31, 32]. Pulijala et al. examined the efficiency of immersive VR in surgical training in novice surgical residents. The results of this study showed that iVR would help maxillofacial surgical technique training (Le Fort I osteotomy). Residents who used this technology showed a higher level of self-confidence and theoretical knowledge [20].

Based on the findings of the present study, VR simulators have a positive effect on improving students' practical skills. The review of studies indicates the effective role of VR in dental surgery education. According to some evidence, manual assessment methods support the superiority of VR-based learning approaches compared to traditional approaches in dental education [18, 22, 25]. The validity of such a result may rise as assessment methods shift to the digital realm, possibly due to a reduction in human-related assessment errors. As shown in Murbay et al. the use of more robust digital assessment methods in ideal settings would better portray the beneficial use of VR technology in operative dental education [18]. In this regard, haptic technology, as a key part of VR technologies, adds a sense of touch to former visual-only interfaces. These systems can simulate tactile input and visual feedback to help the training of clinical psychomotor skills. The ease of use and gaming features of haptic devices can create an interesting learning experience for dental learners [22]. VR technologies also bring navigation systems for teaching the surgical stage of dental implantation. According to the study of Casap et al., students who used this technology showed significantly higher accuracy in marking the first implantation site [25].

## Discussion

This systematic review was conducted to compare VR-based education and conventional learning methods in dental education. For this purpose, the conducted studies were compared regarding the role of VR simulators and conventional learning methods in improving students' theoretical knowledge and practical skills. A systematic review of the conducted studies indicated that VR simulators play an effective role in improving the level of theoretical knowledge and practical skills of dental students. The application of VR in dental education has expanded due to its high potential in overcoming environmental limitations, providing the possibility of frequent training, immediate feedback, real experience in a simulated environment, and educational effectiveness [33, 34].

Each field of dentistry has a distinct educational environment based on its specific characteristics and educational context. As a result, there is a disparity in the applications of VR technologies across various fields of dentistry. VR technologies will become more evenly spread across different fields as time passes and technological limitations are resolved. However, this does not mean that VR applications will take over all areas; rather, they might serve as a complement to traditional learning methods. The ability to provide immediate educational feedback and automatic evaluation are two important features of VR technologies to improve the quality of traditional education [35]. The empirical evidence of some studies indicates that the use of VR simulators along with traditional training creates a favorable approach to teaching dental skills to students [18, 36].

The majority of the studies approve that VR significantly enhances students' practical skills training and proficiency. Despite the consensus on the preferred use of VR systems from the student's perspective, there remains slight controversy about whether or not VR training systems can improve hand skills training and dental students' proficiency [24, 26, 37]. Nevertheless, all studies indicate VR applications are at least as effective as traditional learning methods. The only ambiguity mentioned in the studies is related to the student evaluation methods and the hardware and software used.

VR systems also seem to be valuable in postgraduate dental education, typically with the help of their immersive properties. For instance, novice surgical residents who used the VR surgery training system showed significantly higher self-confidence than the control group. It's also declared that this higher self-confidence is associated with an enhanced comprehensive transfer of knowledge obtained during VR training's multisensory integrated experience [20]. In general, VR simulators provide the possibility of evaluating different areas of the body for the diagnosis, planning, and training of dental surgery and can create tremendous development in this field in the future [38–40]. In line with previous statements, other studies show that the VR groups generally acquired higher knowledge scores than the traditional control groups. In the study of Kyaw et al., VR is counted as one of the primary forms of blended digital education, which has added educational values in communication skills and knowledge transfer [41].

In general, it can be said that today one of the most important competitive indicators in universities is the ability to use new technologies in the teaching and learning process. There is no doubt that VR simulators in dental education can cause a tremendous change in the training of capable and skilled students. VR technologies can be as effective as conventional teaching methods in dental education. These technologies can be a very valuable supplement to conventional training in dental schools. Designing educational scenarios is essential for the effective integration of virtual reality simulators in the educational process.

One of the common limitations in systematic review studies is related to bias in the selection of articles. To overcome this limitation, the search strategy was designed to include all studies in this field. Considering that the included studies had different goals in the use of virtual reality in dental education, it was not possible to conduct a meta-analysis in this study. The number of studies reviewed was small. Nevertheless, these studies show the positive effects of using virtual reality in improving students' learning performance.

## Conclusion

The main goal of this systematic study was to investigate the effectiveness of VR technology in dental education. In this study, conventional education approaches were compared with virtual reality-based education. A comprehensive review of studies indicated that compared to conventional learning approaches, virtual reality-based education plays an effective role in improving the level of theoretical knowledge and practical skills of dental students. In addition, this educational approach can positively affect the level of self-confidence of learners in the learning process and create a more attractive learning environment.

## Data Availability

Data and materials are available by contacting the corresponding author.

## Abbreviations

VR: Virtual reality
VRS: Virtual reality simulation
IVR: Immersive virtual reality
3D: Three dimensional
